# Comparative evaluation of viability assays and functional analysis of *bbi29* in *Borrelia burgdorferi*

**DOI:** 10.1128/aem.01574-26

**Published:** 2026-09-29

**Authors:** Connor Waldron, Sierra George, Zhiming Ouyang

**Affiliations:** 1 Department of Molecular Medicine, University of South Florida 685045, Tampa, Florida, USA; Indiana University Bloomington, Bloomington, Indiana, USA

**Keywords:** transcriptional regulators, gene expression, spirochetes, *Borrelia burgdorferi*, Lyme disease

## Abstract

*In vitro* viability assays are essential for studying the survival of *Borrelia burgdorferi* after exposure to stressful conditions. In contrast to the cumbersome and tedious manual microscopic counting method, fluorescence-based assays are more rapid and convenient and thus can be used in a high-throughput format. In the SYBR Green I/propidium iodide (PI) assay, spirochetes are labeled with different fluorescent dyes, and fluorescence ratios are then compared to calculate the ratio between live and dead spirochetes. Stained spirochetes are also manually counted under a fluorescence microscope or analyzed via flow cytometry to obtain the live/dead ratio. In this study, we compared these methods to determine the most accurate technique for quantifying spirochete viability. Our results showed that, compared with the SYBR Green I/PI assay that carries inherent caveats, both the fluorescent microscopic counting method and the flow cytometry method provide more reliable and accurate results for *B. burgdorferi* viability assays, and flow cytometry offers significant advantages in speed and reduced labor compared to manual microscopic counting. Leveraging the flow cytometry-based viability assay, we investigated the role of gene *bbi29*, encoding a putative virulence-associated lipoprotein, in *B. burgdorferi* stress response. The contribution of *bbi29* to *B. burgdorferi* mammalian infection was also examined using a murine model of Lyme disease.

**IMPORTANCE:**

Lyme disease, caused by *Borrelia burgdorferi*, is the most common tick-borne disease in the United States. Assessment of *B. burgdorferi* growth and viability has been traditionally challenging due to the slow growth and unique morphology of the spirochete. Recently, an optimized SYBR Green I/PI assay was developed and has since been used by a number of laboratories to assess *B. burgdorferi* viability. We herein surprisingly found that the SYBR Green I/PI assay has significant caveats and does not provide accurate quantification of *B. burgdorferi* viability. Rather, our data showed that flow cytometry is a superior alternative to quantify spirochete viability, providing a valuable methodological framework for future studies. Additionally, by investigating the role and requirement for gene *bbi29*, we have refined our understanding of hypothetical proteins in *B. burgdorferi* pathogenesis. Together, this work highlights the importance of developing and validating experimental approaches and functionally characterizing *B. burgdorferi* genes with unknown functions.

## INTRODUCTION

Lyme disease, the most common tick-borne disease in the United States, is caused by *Borrelia burgdorferi* (aka *Borreliella burgdorferi*). In the laboratory, viability assays are commonly performed to determine the susceptibility of *B. burgdorferi* to stressors encountered during its natural enzootic lifecycle. For example, genetically modified spirochetes lacking a specific gene can be exposed to reactive oxygen species (ROS), reactive nitrogen species (RNS), or osmotic stress to investigate the potential role of the gene of interest in responding to these environmental challenges (1–7). Traditionally, assessment of *B. burgdorferi* growth and viability has been challenging. *B. burgdorferi* typically needs 2–3 weeks to form visible colonies on an agar plate for consistent enumeration. Moreover, manual counting of bacterial colonies is cumbersome and tedious. Fluorescence-based assays were developed recently for measuring *B. burgdorferi* viability (8–18). In these assays, live cells with intact membranes are permeable to SYBR Green I, which binds to the minor groove of double-stranded DNA (19–21). Conversely, dead cells with damaged membranes are permeable to propidium iodide (PI), which intercalates double-stranded DNA without sequence preference (22). After staining, live/dead cells can be visualized and enumerated directly using a fluorescence microscope (23, 24). Alternatively, in assays such as the LIVE/DEAD BacLight assay and the SYBR Green I/PI assay, fluorescent ratios are obtained to calculate the percentage of live cells in each sample (8, 23). In particular, the SYBR Green I/PI assay has been used in several studies for high-throughput drug screening and for evaluating the susceptibility of *B. burgdorferi* to antibiotics (11, 13, 15–17). Moreover, fluorescently stained cells can be analyzed by using flow cytometry for quantifying the live/dead ratio (18, 25).

To identify the best method for assessing *B. burgdorferi* viability, we compared these fluorescent-based assays for their capacity to quantify live and dead spirochetes. Our results showed that the SYBR Green I/PI assay has significant caveats and does not provide accurate quantification of *B. burgdorferi* viability. Rather, the flow cytometry method is the most convenient and reliable technique for measuring *B. burgdorferi* viability. As such, we applied this technique and investigated the role of gene *bbi29* in the response of *B. burgdorferi* to reactive oxygen species and osmotic stress. Our previous study reported that expression of *bbi29* is highly regulated by BadR, a transcriptional regulator essential for *B. burgdorferi* infection in mammals (26). Sequence information suggested that *bbi29* encodes a putative virulence-associated lipoprotein, but its exact role in *B. burgdorferi* pathogenesis remains unknown. The requirement for *bbi29* during mammalian infection was thus also examined by using an *in vivo* bioluminescence imaging model.

## RESULTS

### The SYBR Green I/PI assay does not accurately identify live and dead spirochetes due to high background fluorescence

**Fig 1:**
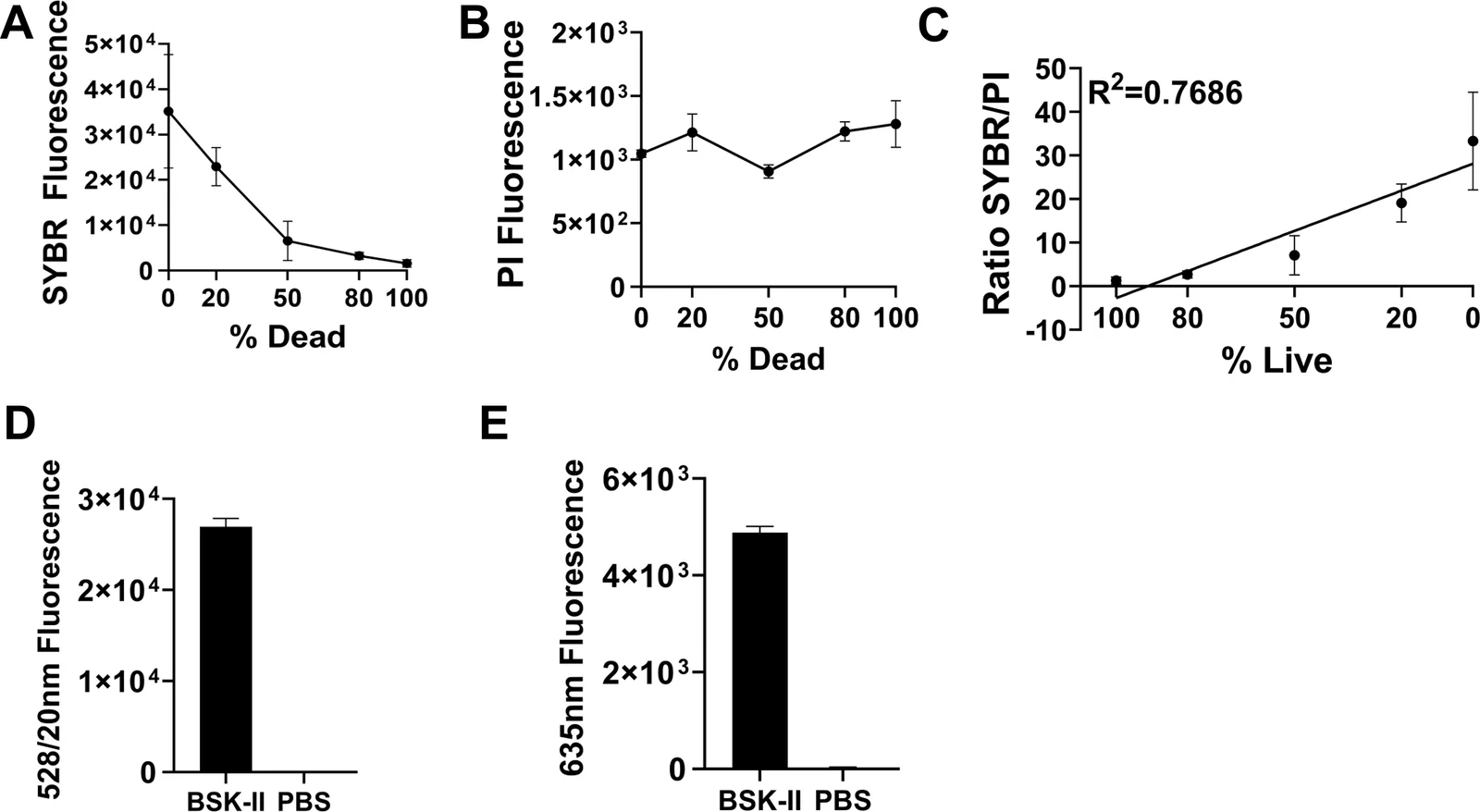
Detection of SYBR Green I and PI fluorescence as measured by the SYBR Green I/PI microplate assay. Standard samples containing proportional live and dead cells (i.e., heat-killed) were prepared as described in the text. (**A and B**) Individual fluorescence values for SYBR Green I (**A**) and PI (**B**) were measured from suspensions of live and dead spirochetes. (**C**) The SYBR/PI ratio was calculated, and a simple linear regression was performed using GraphPad Prism 10.6.1. (**D and E**) Background fluorescence at 528/20 nm (**D**) and 635 nm (**E**) was measured in BSK-II and PBS. Data are presented as the mean values ± standard deviation from three biological replicates.

To compare methods for assessing *B. burgdorferi* viability, we first prepared a series of standard samples by mixing live and dead spirochetes in various proportions (e.g., 0%, 20%, 50%, 80%, and 100%). More specifically, spirochetes were collected from mid-logarithmic phase culture (~5 × 10^7^ spirochetes per mL) via centrifugation, washed, and suspended in 1× phosphate-buffered saline solution (PBS). Samples containing proportional live and heat-killed dead cells were then stained with a dye mixture containing both SYBR Green I and PI as described in the SYBR Green I/PI assay (8), and live and dead cells in each sample were analyzed. In the SYBR Green I/PI assay developed by Feng et al. (8), a linear relationship was found between *B. burgdorferi* viability and the green/red fluorescence ratio, and the regression equation of the standard curve was used to calculate the percentage of live cells in each sample. Implementing the same protocol, we measured the green/red fluorescence ratios of those standard samples and plotted them against the percentage of live cells. Because the SYBR Green I dye stains both live and dead cells, its fluorescence intensity should theoretically remain constant across samples with varying percentages of live and dead cells. In contrast, the SYBR Green I fluorescence decreased as the percentage of dead cells increased from 0% to 100% (Fig. 1A). In a sample containing 0% dead cells, PI fluorescence would be expected to be minimal since PI stains only dead cells. However, PI fluorescence was detected at approximately 1 × 10^3^ RFU in the 0% dead mixture and increased only marginally with increasing proportions of dead cells (Fig. 1B). To further assess the relationship between fluorescence ratios and percentage of live cells, a linear regression analysis was performed. The comparison yielded an R² value of 0.7686 (Fig. 1C), indicating a weak correlation between the percentage of live bacteria and the SYBR/PI fluorescence ratio.

In the study that developed the optimized SYBR Green I/PI assay, *B. burgdorferi* culture (~1 × 10^6^ spirochetes) along with the BSK medium was transferred into a 96-well tissue culture microplate, and the dye mixture was then added to each well before fluorescence measurement (8). In this study, we also added cultures containing ~5 × 10^7^, 5 × 10^6^, or 5 × 10^5^ spirochetes into a 96-well plate and then added the staining mixture to each well. However, upon fluorescence measurement, we observed that SYBR Green I fluorescence was excessively high, resulting in signal saturation and an “overflow” reading (data thus could not be quantified). Such a signal was not observed when spirochetes were resuspended in PBS (Fig. 1A and B), suggesting that the “overflow” fluorescence may originate in part from the BSK medium. To test this hypothesis, we first mixed only the BSK-II medium with the staining dyes and measured fluorescence. As a result, an “overflow” reading was also observed. Next, we measured fluorescence emitted from BSK-II without staining dyes at 528/20 nm and 635 nm, the same emission spectra used to measure SYBR Green I and PI, respectively. The observed fluorescent readings were approximately 2.5 × 10^4^ at 528/20 nm and 5 × 10^3^ at 635 nm, indicating that BSK-II alone emits significant fluorescence (Fig. 1D and E). Conversely, PBS alone did not show significant fluorescence signals (Fig. 1D and E). Collectively, these findings demonstrate that the SYBR Green I/PI assay produces unreliable fluorescence measurements, likely due to high background signal, and is therefore not a suitable method for distinguishing live and dead spirochetes.

### Fluorescent microscopic counting is comparable to flow cytometry for quantifying live and dead spirochetes

**Fig 2:**
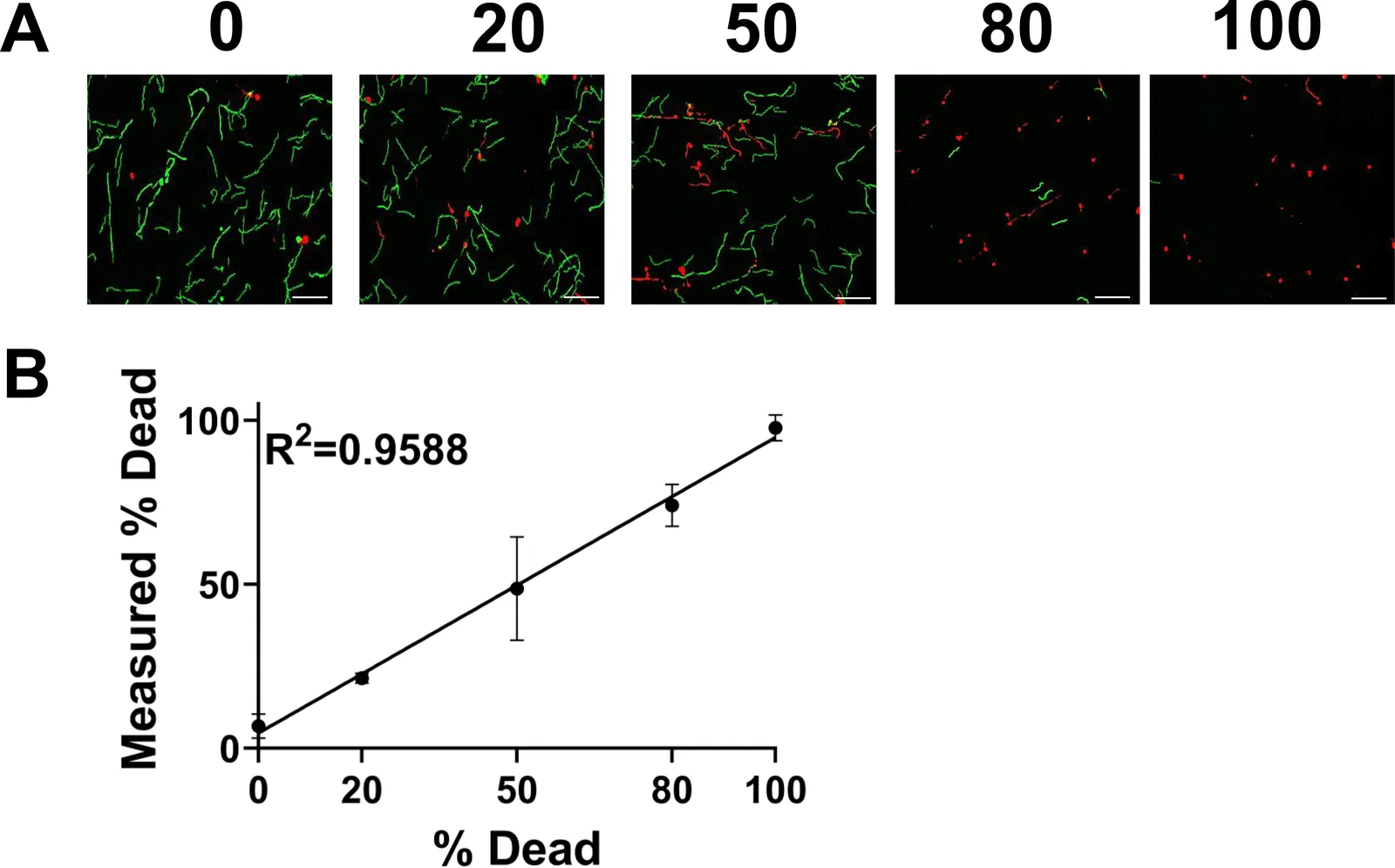
*B. burgdorferi* viability measured by fluorescent microscopy counting. (**A**) Fluorescent microscopy images from suspensions of live and heat-killed spirochetes after staining with PI (red) and SYBR Green I (green). A single representative image is shown from three biological replicates. Numbers above the images show the percentage of dead cells in the standard samples. Scale bars: 10 µm. (**B**) Percentages of dead spirochetes were measured by fluorescence microscopy and plotted against the known percentage of dead spirochetes per sample. Simple linear regressions were performed using GraphPad Prism 10.6.1. Data are presented as the mean values ± standard deviation from three biological replicates.

As the fluorescence ratio did not correlate linearly with the percentage of live cells, we used fluorescence microscopy to examine the same standard samples. Images were acquired, and green or red spirochetes were manually counted. As shown in Fig. 2A, percentages of dead spirochetes measured by fluorescence microscopy counting were 6.8%, 21.4%, 48.7%, 74.4%, and 97.7%, respectively, in the standard samples. When directly compared using a linear regression, actual percentages compared to measured percentages of dead spirochetes resulted in an excellent linear correlation (*R*^2^ = 0.9588), suggesting that fluorescent microscopic counting is an accurate method to quantify live and dead spirochetes (Fig. 2B).

**Fig 3:**
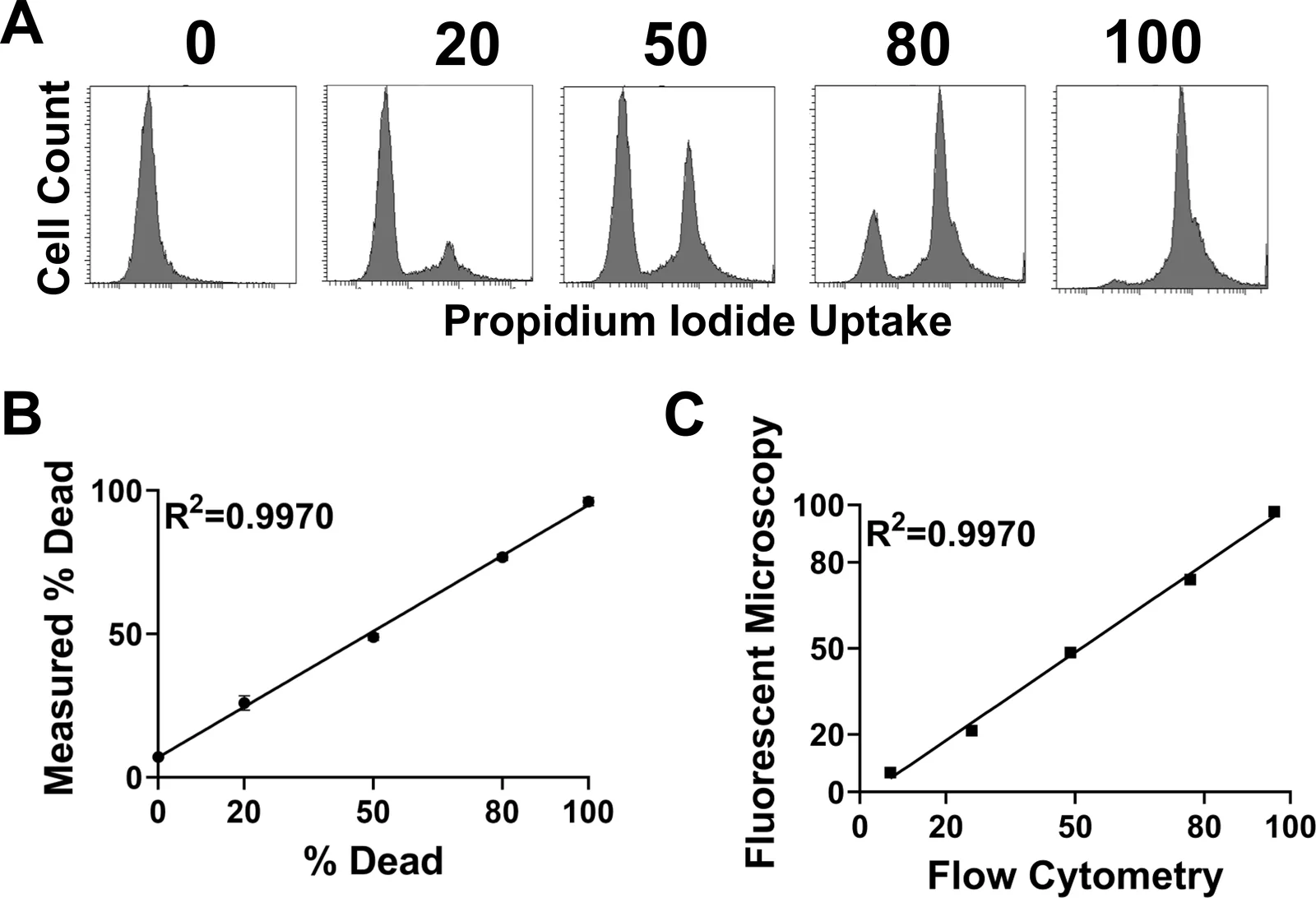
*B. burgdorferi* viability measured by flow cytometry. (**A**) Flow cytometry histograms from suspensions of live and heat-killed spirochetes after staining with PI. A single representative image is shown from three biological replicates. (**B**) Percentages of dead spirochetes were measured by flow cytometry and plotted against the known percentage of dead spirochetes per sample. (**C**) Correlation between measured percent dead values using fluorescent microscopy and flow cytometry. Simple linear regressions were performed using GraphPad Prism 10.6.1. Data are presented as the mean values ± standard deviation from three biological replicates.

Flow cytometry has been used widely in bacterial viability assays (27–32). Therefore, we also used flow cytometry to quantify live and dead cells in the SYBR/PI-stained standard samples. However, overlap in the emission spectra of SYBR Green I and PI can lead to spillover, whereby SYBR fluorescence is detected on the PI channel in flow cytometry (33). Moreover, PI alone was used previously in flow cytometry for quantifying live/dead spirochetes (25). As such, SYBR Green I was excluded from the flow cytometry protocol performed here. Specifically, spirochetes were collected at the mid-logarithmic phase and subsequently subjected to analysis. Gating strategies for flow cytometry were described in Fig. S1. As shown in Fig. 3A, flow cytometry measured 7.1%, 26.0%, 48.9%, 76.8%, and 96.2% dead spirochetes in standard samples containing 0%, 20%, 50%, 80%, and 100% dead spirochetes. Linear regression of flow cytometry results compared to known percentages of dead spirochetes resulted in an *R*^2^ of 0.9970, indicating a strong correlation (Fig. 3B). Together, these results confirm that flow cytometry is an accurate method for quantitating live and dead spirochetes.

Data gained from the fluorescent microscopic counting method and the flow cytometry method were directly compared using an attribution plot. As shown in Fig. 3C, the results from both methods showed an excellent correlation (*R*^2^ = 0.9970), suggesting that they are comparable for identifying and quantifying live and dead spirochetes. We also prepared live/heat-killed standard samples using spirochetes collected at the late-logarithmic growth phase (~1–2 × 10^8^ spirochetes per mL). When those samples were analyzed by fluorescent microscopic counting and flow cytometry, similar results were obtained (Fig. S2).

In the SYBR Green I/PI assay described by Feng et al*.* (8), spirochetes were treated with 70% isopropyl alcohol to obtain dead cells. We also tried to kill spirochetes using 30%, 50%, or 70% isopropyl alcohol. However, such efforts failed after multiple attempts. Once the spirochete pellet was mixed with isopropyl alcohol, spirochetes clustered together and could not be separated and resuspended. In nature, *B. burgdorferi* encounters reactive oxygen species upon transmission from ticks to mammals in the tick saliva (34, 35). Additionally, phagocytic cells expose *B. burgdorferi* to ROS after phagocytosis (36). Therefore, exposure to H_2_O_2_ also constitutes a natural condition that produces dead spirochetes. Previously, Hyde et al. (25) found that ~90% of spirochetes were dead when treated with 50 mM H_2_O_2_ for 4 h. We used this treatment to obtain dead cells and found that spirochetes were all dead when treated with 100 mM H_2_O_2_ for 4 h. Live/dead standard samples were then prepared and analyzed by both fluorescent microscopic counting and flow cytometry. Consistent with data obtained for live/heat-killed spirochetes, our data (Fig. S3 and S4) support that both methods are valid for quantifying live/H_2_O_2_-killed spirochetes, suggesting that the killing method does not affect the assays.

### BBI29, a putative virulence-associated lipoprotein, is not required by *B. burgdorferi* to infect mammals

Previous studies have demonstrated that the transcriptional regulator BadR regulates expression of the alternative sigma factor RpoS and is essential for *B. burgdorferi* infection in mammalian hosts (26, 37–39). Gene expression analyses supported that BadR is a global transcriptional regulator and modulates the expression of numerous hypothetical proteins, including BBI29 (26, 37). Specifically, previous RNA sequencing from our lab showed that expression of *bbi29* is downregulated ~10-fold in a *badR* mutant, which was substantiated by qRT-PCR analysis (26). Moreover, expression of *bbi29* was shown to be affected by temperature changes, with significantly higher transcription levels at 35°C compared to 23°C (40). Sequence analysis showed that the amino acid sequence of BBI29 is more than 99.5% conserved among *B. burgdorferi* clinical isolates. *bbi29* was originally predicted to encode a conserved hypothetical protein with unknown function but is currently annotated as encoding a putative virulence-associated lipoprotein in *B. burgdorferi* (41). Further analysis using the NCBI Conserved Domain Search identified a putative BbCRASP-1 (*B. burgdorferi* complement regulator-acquiring surface protein 1) domain in BBI29. BbCRASP-1 domains are also found in other *B. burgdorferi* proteins, including BBA68 (CspA), BBE31, BBA66, and BBA69. However, sequence alignments of BBI29 to CspA, BBE31, BBA66, and BBA69 identified only ~18%, 31%, 21%, and 17% sequence similarity, respectively (Fig. S5). Moreover, only one of the 13 residues important for binding of CspA to host complement factors is conserved in BBI29 (Fig. S5) (42). In addition, the structure of BBI29 was modeled here using AlphaFold V2.0 DB version 2022-05-31 and compared with the crystal structures of CspA, BBE31, BBA66, and BBA69 (43–46). As shown in Fig. S6, structural comparison of BBI29 to CspA, BBE31, BBA66, and BBA69 resulted in RMSD scores of 9.574, 1.997, 6.891, and 5.965, respectively.

**Fig 4:**
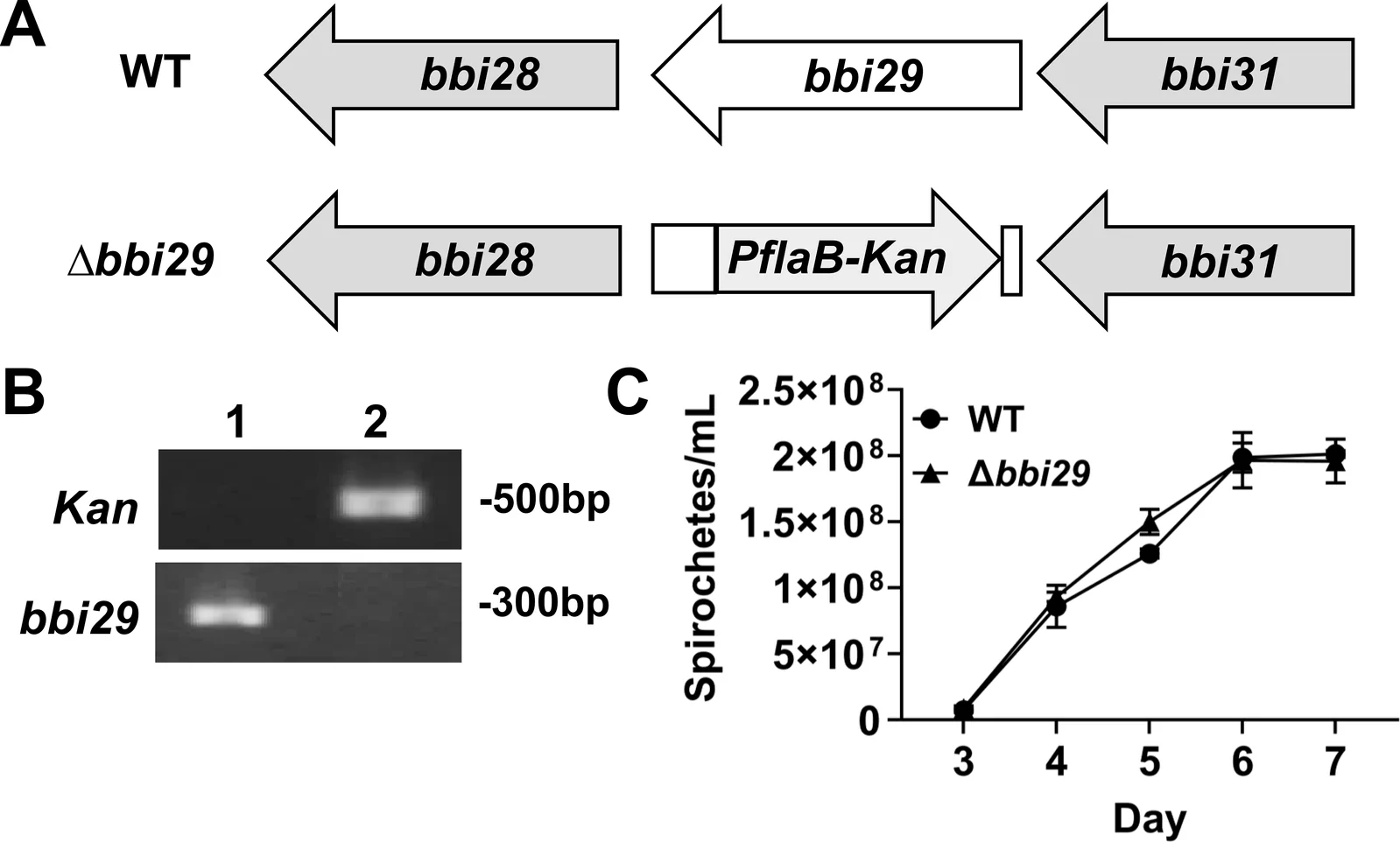
Inactivation of *bbi29* in *B. burgdorferi*. (**A**) Schematic representation of the *bbi29* locus in WT OY617 and the *bbi29* mutant. Genes are shown as thick arrows circumscribing the respective gene numbers. (**B**) Strains were confirmed by isolating gDNA and performing PCR with primers targeting *bbi29* and the kanamycin resistance gene. Lane 1, WT; Lane 2, the *bbi29* mutant. (**C**) Growth phenotypes of WT and the *bbi29* mutant in BSK-II at 37°C. Spirochetes were enumerated daily by dark-field microscopy. Data are presented as the mean values ± standard deviation from three biological replicates. The mean values between WT and the mutant were compared using the Student’s *t*-test and were not significantly different (*P* > 0.05).

**Fig 5:**
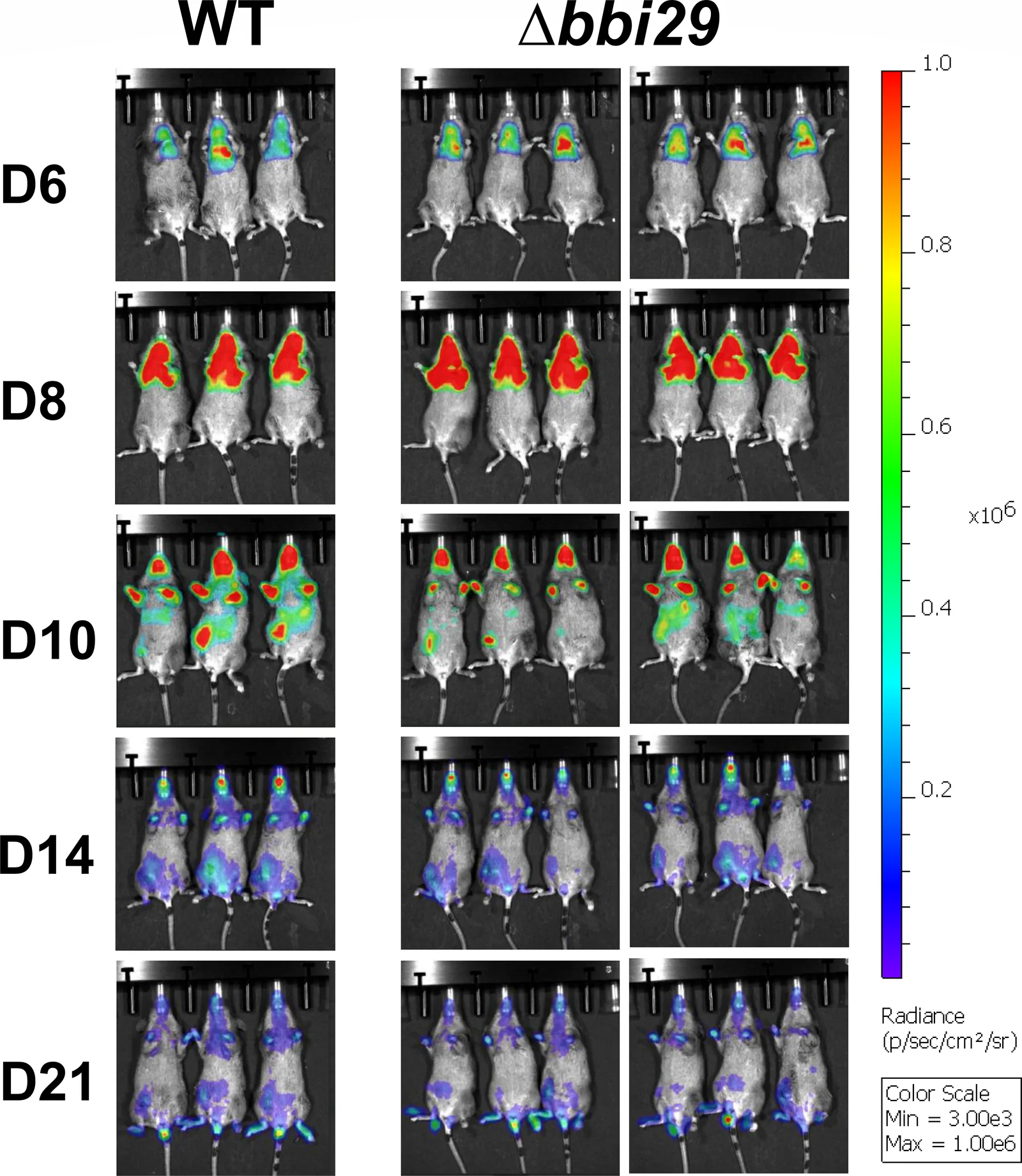
Bioluminescence imaging of *B. burgdorferi* in the murine model. Groups of C3H/HeN mice were injected intradermally with WT OY617 and the *bbi29* mutant (∆*bbi29*) at a dose of 10,000 spirochetes per mouse. On days 6, 8, 10, 14, and 21 post-infection, mice were injected with D-luciferin and analyzed using a PerkinElmer IVIS imaging system. All images are normalized to the same p/s range and displayed on the same color spectrum scale (right). D, day.

To experimentally investigate the role of BBI29 during *B. burgdorferi* mammalian infection, a *bbi29* mutant was created in a bioluminescent, infectious *B. burgdorferi* strain OY617 (Fig. 4A). Using PCR, the *bbi29* mutant was confirmed to lack the gene *bbi29* and carry the kanamycin resistance cassette (Fig. 4B). PCR-based plasmid profiling indicated that the mutant retains all essential endogenous plasmids (e.g., lp17, lp25, lp26, lp28-1, lp36, and lp54) contained in wild type (WT) OY617 (Fig. S7). No obvious differences were observed between WT OY617 and the *bbi29* mutant when spirochete morphology was examined using dark-field microscopy. The effect of the inactivation of *bbi29* on *B. burgdorferi in vitro* growth was assessed by determining the growth curves of strains grown in BSK-II medium. As shown in Fig. 4C, no discernible effect on growth was observed when *bbi29* was inactivated, and the *bbi29* mutant displayed similar growth to WT. The infectivity of the *bbi29* mutant was tested using a murine model of Lyme disease. When C3H mice were injected with 10,000 WT spirochetes, bioluminescence was detected beginning on day 6, followed by peak bioluminescence on day 8, and a period of spirochete dissemination and bacterial clearance on days 10, 14, and 21 post-injection. Interestingly, C3H mice injected with 10,000 *bbi29* mutant spirochetes exhibited a similar infection pattern to WT-infected mice (Fig. 5). Furthermore, spirochetes were recovered from all injection skin, distal skin, joints, and heart tissues harvested from WT- and *bbi29* mutant-infected mice (Table 1). Combined, these results indicate that BBI29 is not essential for *B. burgdorferi* to infect mammals.

**TABLE 1:** *B. burgdorferi* recovery from mouse tissues^a^

Strain	Dose	No. of cultures positive/total no. of specimens examined					No. of mice infected/total no. of mice
		Injection skin	Distal skin	Joint	Heart	All sites	
WT	10^4^	3/3	3/3	3/3	3/3	12/12	3/3
∆*bbi29*	10^4^	6/6	6/6	6/6	6/6	24/24	6/6

^*a*^ Injection skin (skin collected from the site of injection), distal skin (skin collected from the distal back), joint, and heart tissues were collected from C3H mice at 21 days after infection with *B. burgdorferi* WT OY617 or the *bbi29* mutant (∆*bbi29*). Tissues were cultured in BSK-II medium, and spirochete recovery was determined by observing spirochetes under dark-field microscopy.

### *bbi29* is not involved in *B. burgdorferi* response to H_2_O_2_ or NaCl

**Fig 6:**
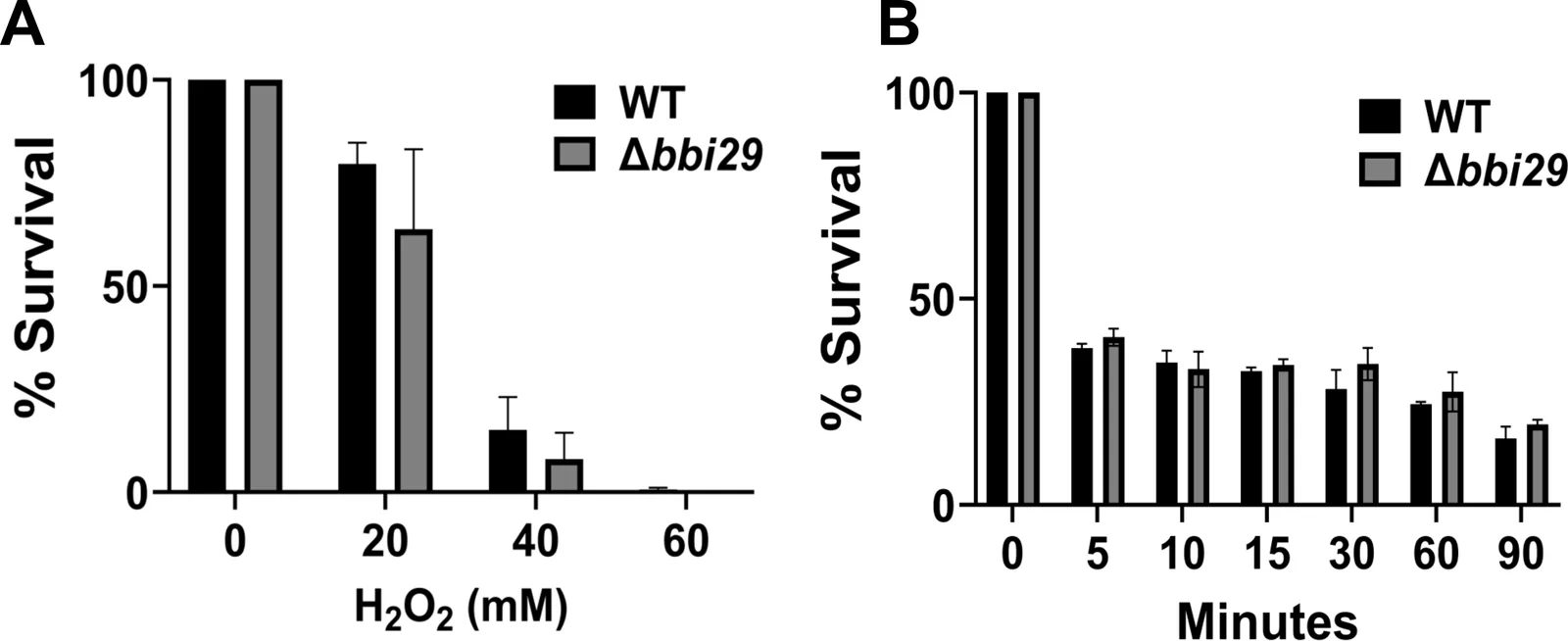
*bbi29* is not required for *B. burgdorferi* response to ROS or osmotic stress. WT OY617 and the *bbi29* mutant (∆*bbi29*) were challenged with (**A**) increasing concentrations of H_2_O_2_ and (**B**) 1 M NaCl. Spirochete viability was determined by using flow cytometry, and percent survival was calculated. Data are presented as the mean values ± standard deviation from three biological replicates. The mean values between WT and the mutant were compared using the Student’s *t*-test and were not significantly different (*P* > 0.05).

To survive in ticks and mammals, *B. burgdorferi* must overcome various environmental stresses such as osmotic pressure and oxidative stress (34, 35, 47, 48). To date, such mechanisms remain largely unknown. To investigate whether *bbi29* contributes to the stress response of *B. burgdorferi*, the sensitivity of WT and the *bbi29* mutant to ROS and osmotic stress was compared using fluorescence-based assays coupled with flow cytometry. In brief, the parental strain OY617 and the *bbi29* mutant were grown in BSK-II medium. When cells reached the mid-logarithmic phase of growth, ~5 × 10^7^ cells were collected, exposed to H_2_O_2_ or NaCl, stained with PI, and analyzed by flow cytometry. As shown in Fig. 6, there was no significant difference in the survival rate between WT and the *bbi29* mutant after exposure to H_2_O_2_ and NaCl, suggesting that *bbi29* does not contribute to the resistance of *B. burgdorferi* to ROS or osmotic stress.

## DISCUSSION

To identify a reliable and rapid method for assessing *B. burgdorferi* viability, we compared three fluorescence-based assays for assessing *B. burgdorferi* viability. We first implemented the SYBR Green I/PI assay to test standard samples prepared by mixing live and dead spirochetes. Surprisingly, different from the report by Feng et al. (8), our data did not show a strong correlation between the SYBR/PI ratio and the percentage of live cells (Fig. 1A through C). Moreover, high background fluorescence was observed from BSK-II medium (Fig. 1D and E). Together, these observations suggest that the SYBR Green I/PI assay may not be a reliable method to provide accurate results for assessing *B. burgdorferi* viability. Flow cytometry has been used to study the viability of many bacteria, including *E. coli*, *Pseudomonas aeruginosa*, *Staphylococcus aureus*, *Helicobacter pylori*, and, more recently, *B. burgdorferi* (18, 25, 29–32, 49, 50). Therefore, we also used flow cytometry for enumerating fluorescently stained spirochetes. Live/dead cells were also directly visualized and enumerated using the fluorescent microscopic counting method. Our data showed that both methods provided accurate and comparable results for quantifying cell viability for spirochetes collected at the mid-logarithmic and late-logarithmic phases of growth (Fig. 2 and 3; Fig. S1 to S4). Notably, flow cytometry is faster and less laborious than fluorescent microscopic counting. We anticipate flow cytometry will be a useful tool for investigating other morphological characteristics of *B. burgdorferi*, including cell size, length, and pleomorphic forms.

After establishing the accuracy and reliability of flow cytometry for studying cell viability, we applied the method to investigate the contribution of *B. burgdorferi* genes dedicated to bacterial response to environmental stresses such as ROS and osmotic stress. To this end, we selected *bbi29* for further analysis based on several lines of evidence. First, *bbi29* is well conserved in clinical isolates of *B. burgdorferi*. This gene is encoded by the endogenous plasmid lp28-4, a plasmid reportedly important for murine infection by Lyme *Borrelia*, including *B. burgdorferi* and *B. mayonii* (51–54). Moreover, expression of *bbi29* is highly regulated by BadR, a transcriptional regulator essential for *B. burgdorferi* mammalian infection (26). Additionally, *in silico* analysis suggested that *bbi29* encodes a putative virulence-associated factor, but its exact role remains unknown. Therefore, we hypothesized that BBI29 may be important during *B. burgdorferi* mammalian infection. However, upon *in vivo* bioluminescence imaging, C3H mice that received an injection of WT or the *bbi29* mutant exhibited similar infection dynamics (Fig. 5). When exposed to ROS or osmotic stress, no significant difference in bacterial survival was observed between WT and the *bbi29* mutant (Fig. 6A and B). While a simple interpretation of our data is that BBI29 may not be essential for *B. burgdorferi* stress responses or infectivity, they do not exclude the possibility that BBI29 may play a role in infection. The current study examined *B. burgdorferi* infectivity only in a widely accepted murine model, and BBI29 may be important for *B. burgdorferi* infection and pathogenesis in other animals. Moreover, the mutant may exhibit impaired infectivity in animals following tick transmission. In addition, BBI29 may contribute to the survival and persistence of *B. burgdorferi* in ticks, which is buttressed by the observation that *bbi29* was highly expressed in fed larvae and fed nymphs (55). These possibilities will be addressed in the future to investigate the role of this protein during the complete tick-mammal infectious cycle. To date, the biological function of BBI29 in *B. burgdorferi* also remains unknown. Initial sequence analysis implicated BBI29 as a PFam54-60 family protein with a putative C-terminal BbCRASP-1 domain; BbCRASP-1 binds to human complement regulators and thus may play a critical role in complement evasion. However, further sequence and structural comparison of BBI29 to other BbCRASP-1 domain-containing proteins resulted in low similarity and low RMSD scores (Fig. S5 and S6). It is thus likely that BBI29 does not function as a BbCRASP-1 protein and instead plays a unique, yet to be identified, role in *B. burgdorferi*. Future work is thus warranted to elucidate the molecular function of this protein.

## MATERIALS AND METHODS

### Bacterial strains and growth conditions

*B. burgdorferi* were routinely grown in Barbour-Stoenner-Kelly-II medium (BSK-II) supplemented with 6% heat-inactivated rabbit serum at 37°C and 5% CO_2_. Kanamycin (320 μg/mL) or streptomycin (100 μg/mL) were added to BSK-II as needed. Spirochetes were enumerated by dark-field microscopy using a Petroff-Hausser counting chamber to determine cell density. *Escherichia coli* strain Top10, used as the cloning host in this study, was grown in liquid LB medium or on solid LB agar plates supplemented with kanamycin (50 μg/mL), as appropriate. All recombinant plasmids were analyzed by using PCR, restriction digestion, and DNA sequencing.

A series of mixtures containing known proportions of live and dead spirochetes were prepared as standards in viability assays. Specifically, *B. burgdorferi* strain 297 (56) was grown in BSK-II at 37°C, and spirochetes were collected by centrifugation when growth reached the mid-logarithmic (~5 × 10⁷ spirochetes per mL) or late-logarithmic (~1–2 × 10^8^ spirochetes per mL) growth phase. Cells were washed thrice, resuspended in 1× PBS to a final concentration of 5 × 10^8^ spirochetes per mL, and divided into two groups: dead and live cells. Dead cells were heat-killed, as previously described (14, 36, 57, 58), by incubating live spirochetes in a 55°C water bath for 30 min or incubating with 100 mM H_2_O_2_ for 4 h at 37°C. Live cells were then mixed with dead cells at defined ratios of 10:0, 8:2, 5:5, 2:8, and 0:10, respectively, generating samples containing known percentages of dead spirochetes (0%, 20%, 50%, 80%, and 100% dead). Cell suspensions were incubated with a dye mixture containing 144× SYBR Green I (Invitrogen) and 0.5 mM propidium iodide (PI) (Alfa Aesar) for 15 min in the dark, as previously described (27, 28). Live/dead cells were quantified using the SYBR Green I/PI assay or fluorescent microscopic counting.

### The SYBR Green I/PI assay

The SYBR Green I/PI assay was performed as previously described (8). In brief, stained cells were transferred to a Costar 96-well assay microplate, and fluorescence was measured on a Synergy H4 microplate reader. Relative fluorescence units (RFUs) were measured at excitation/emission wavelengths of 485/528 nm for SYBR Green I and 485/635 nm for PI. A blank measurement consisting of 1× PBS was included, and background fluorescence was subtracted from all raw RFU values prior to analysis. Percent viability was calculated as the ratio of SYBR Green I fluorescence to PI fluorescence. Ratios were plotted as a simple linear regression, and *R*^2^ values were calculated using GraphPad Prism 10 V10.6.1.

### Fluorescent microscopy

Fluorescent microscopy was performed using a BZ-X710 Keyence fluorescence microscope equipped with green and red filters to visualize SYBR Green I and PI, respectively (59). Five images were taken at random per sample, and ~80 cells were manually counted per image. Percent viability was calculated as the ratio of SYBR Green I-stained (green, live) cells to PI-stained (red, dead) cells. Ratios were plotted as a simple linear regression, and *R*^2^ values were calculated using GraphPad Prism 10 V10.6.1.

### Flow cytometry

For each sample, ~5 × 10^7^ cells were collected, resuspended in 1× PBS, and incubated in the dark for 15 min with 35 μM PI (Alfa Aesar). Following staining, cells were centrifuged and resuspended in 1× PBS prior to analysis. A sample without PI was included as a negative control. Flow cytometry was performed in triplicate using a BD FACSCanto II cytometer. Data were acquired and analyzed using BD FACSCanto software (version 6.1.3). Forward scatter (FSC) and side scatter (SSC) parameters were used to gate events corresponding to cells of appropriate size and granularity. Voltages for the FSC and SSC channels were 775 V and 720 V, respectively. A threshold of 11,000 was applied to FSC, and 5,000 was applied to SSC. The PI signal was measured on a 488-nm laser with a voltage of 489 V. Rectangle gates were plotted on SSC vs PI contour plots; the resulting dependent population was then plotted on histograms, and PI-positive events were gated via an interval gate. Dead cells were identified as events exhibiting PI fluorescence intensity greater than 10^3^. A total of 50,000 events were collected per sample.

### Construction of the *bbi29* mutant

To investigate the requirement for *bbi29* during mammalian infection using an *in vivo* bioluminescence model, a strain constitutively expressing luciferase was generated in the B31-A3-68 background, as previously described (60). In brief, the P*flaB-Bbluc* cassette was integrated into the intergenic region between *bbb20* and *bbb21* by homologous recombination. The resulting strain, OY617, was used as the parental strain to create the *bbi29* mutant. To create the *bbi29* mutant, an 874-bp fragment upstream of *bbi29* and a 971-bp fragment downstream of *bbi29* were PCR-amplified from *B. burgdorferi* using primer pairs CW04.1F/CW04.1R and CW03.1F/CW03.1R, respectively (1, 4, 60, 61). These two DNA fragments were cloned and assembled together with the kanamycin resistance cassette and the pUC19 origin of replication (1–3, 61) using the GeneArt Seamless Cloning and Assembly Kit (Thermo Fisher Scientific) and *E. coli* Top10. The resulting suicide plasmid, pCW04, was transformed into *B. burgdorferi* strain OY617 by electroporation, generating strain CW03. This strategy replaced a 493-bp coding region of *bbi29* with the kanamycin cassette. Positive transformants were selected using kanamycin and confirmed using PCR. Plasmid content of the mutant was analyzed by multiplex PCR (62).

### ROS and osmotic shock assays

Resistance of spirochete to reactive oxygen species (ROS) and osmotic stress was analyzed, as previously described (1–4, 60, 63, 64). In brief, *B. burgdorferi* WT strain OY617 and the *bbi29* mutant were grown in BSK-II at 37°C with 5% CO_2_. When cultures reached mid-logarithmic phase (~5 × 10^7^ spirochetes per mL), cells were harvested by centrifugation. Cells were washed in 1× PBS and resuspended to a final concentration of 5 × 10^8^ spirochetes per mL in modified BSK-II medium lacking pyruvate and heat-inactivated rabbit serum; previous reports have shown that extracellular pyruvate protects spirochetes from H_2_O_2_ killing (65). For ROS assays investigating the susceptibility of a *bbi29* mutant to ROS, H_2_O_2_ was added to cell suspensions at final concentrations of 20, 40, or 60 mM and incubated at 37°C for 4 h (1, 2, 4, 63). A negative control group without H_2_O_2_ was included. Following treatment, cells were washed thrice with 1× PBS and prepared for flow cytometry as described above. For osmotic shock assays, NaCl was added to cell suspensions to a final concentration of 1 M and incubated at 37°C (1, 4). After 0-, 5-, 10-, 15-, 30-, 60-, and 90-min incubations, cells were collected by centrifugation, washed thrice with 1× PBS, and prepared for flow cytometry. Percent survival was calculated by dividing the number of spirochetes surviving in the treated group by the number of spirochetes surviving in the control group.

### *In vivo* bioluminescent imaging

To investigate the requirement for *bbi29* during *B. burgdorferi* infection, *in vivo* bioluminescent imaging was performed as previously described (1, 66). Groups of three C3H/HeN mice were injected intradermally with 1 × 10^4^ WT OY617 or the *bbi29* mutant spirochetes. On days 6, 8, 10, 14, and 21 post-infection, mice were injected intraperitoneally with 150 mg/kg of body weight of D-luciferin and imaged on an IVIS Spectrum live imaging system at the University of South Florida. Aura v4.5.1 software was used to collect and analyze all images. Bioluminescence was measured as units of photons/s. Upon completion of the imaging experiment on day 21 post-infection, mice were euthanized, and injection skin (i.e., skin at the injection site), distal skin, joint, and heart were harvested and cultured in BSK-II at 37°C with 5% CO_2_. Cultures were observed by dark-field microscopy for the presence of spirochetes.

### Bioinformatic analysis

Sequence analysis was performed using Clustal Omega v2.10.1, and the output was analyzed via pyBoxshade v1.2 (67) (https://github.com/mdbaron42/pyBoxshade). NCBI Conserved Domain Search was used to identify putative functional domains in BBI29 (68). The crystal structures of the BbCRASP-1 domain proteins BBA68 (CspA) (PDB 1W33), BBA69 (PDB 6QO1, Chain A), BBE31 (PDB 6FZE, Chain A), and BBA66 (PDB 5IZT) were acquired from the PDB database. AlphaFold (69, 70) was used to predict the structure of BBI29. Structure alignments were performed, and RMSD scores were calculated using PyMOL v4.6.0.

### Statistical analyses

Unpaired Student’s *t*-test or one- or two-way analysis of variance (ANOVA) followed by the Tukey test for multiple comparisons was used as appropriate. *P*  ≤  0.05 was used to determine statistical significance. All statistical analyses were performed using GraphPad Prism version 10.6.1.

## SUPPLEMENTAL MATERIAL

Supplemental material aem.01574-26-s0001.docx Fig. S1 to S7; Table S1.

ASM does not own the copyrights to Supplemental Material that may be linked to, or accessed through, an article. The authors have granted ASM a non-exclusive, world-wide license to publish the Supplemental Material files. Please contact the corresponding author directly for reuse.
