# Supplementary material for: Comparative evaluation of viability assays and functional analysis of bbi29 in Borrelia burgdorferi
Source: Appl Environ Microbiol. 2026 Sep 29;92(10):e01574-26. doi: 10.1128/aem.01574-26 (PMC13689468; doi:10.1128/aem.01574-26)
Supplement: Supplementary file 1 [file aem.01574-26-s0001.docx]

**Supplementary Information**

**Comparative evaluation of viability assays and functional analysis of *bbi29* in *Borrelia burgdorferi***

Connor Waldron†, Sierra George†, and Zhiming Ouyang*

Department of Molecular Medicine, University of South Florida, 12901 Bruce B Downs Blvd, MDC 07, Tampa, FL 33612, USA

†These authors contributed equally to this work.

*Correspondence: zouyang@usf.edu; Tel.: 813-974-3325; Fax: 813-974-7357.

Running Title: *Borrelia* viability assays

**Fig. S1. Gating strategies for flow cytometry.**

**Fig. S2. Mixtures containing live and heat-killed late-logarithmic *B. burgdorferi* were analyzed by fluorescent microscopic counting and flow cytometry.**

**Fig. S3. Mixtures containing live and H_2_O_2_-killed *B. burgdorferi* were analyzed by fluorescent microscopy counting.**

**Fig. S4. Mixtures containing live and H_2_O_2_-killed *B. burgdorferi* were analyzed by flow cytometry.**

**Fig. S5. Sequence alignment of BBI29 to other BbCRASP-1 domain containing proteins.**

**Fig. S6. Structural comparison of BBI29 and BbCRASP-1 domain containing proteins.**

**Fig. S7. Plasmid content of *B. burgdorferi* was determined using multiplex PCR.**

**Table S1. Oligonucleotide primers used in this study.**

**Fig. S1. Gating strategies for flow cytometry.** *B. burgdorferi* grown in BSK-II was collected when growth reached the mid-logarithmic phase. Cells were treated via heating at 55°C for 30 minutes to obtain dead cells. Samples containing 50% live cells and 50% dead cells were analyzed by flow cytometry. (A) Forward scatter (FSC-H) *vs*. side scatter (SSC-H) was plotted to represent the distribution of cells size and granularity. (B) Rectangle gate on an SSC-H *vs*. propidium iodide (PI-H) contour plot to first identify populations of spirochete size, shape, and granularity and second sub-populations of PI positive and negative spirochetes. (C) A histogram depicting PI intensity in the gated population.

**Fig. S2. Mixtures containing live and heat-killed late-logarithmic *B. burgdorferi* were analyzed by fluorescent microscopic counting and flow cytometry*.*** *B. burgdorferi* grown in BSK-II was collected when growth reached the late-logarithmic phase. Samples were prepared by mixing proportional live and dead cells. Percentages of dead spirochetes were measured by fluorescent microscopy (A) or flow cytometry (B) and plotted against the known percentage of dead spirochetes per sample. (C) Correlation between measured percent dead cells using fluorescent microscopy and flow cytometry. Simple linear regressions were performed using Graphpad Prism 10.6.1. Data are presented as the mean values ± standard deviation from three biological replicates.

**Fig. S3. Mixtures containing live and H_2_O_2_-killed *B. burgdorferi* were analyzed by fluorescent microscopy counting.** *B. burgdorferi* grown in BSK-II was collected when growth reached the mid-logarithmic phase. Cells were treated with 100 mM H_2_O_2_ for 4h to obtain dead cells. Samples were prepared by mixing proportional live and dead cells. After staining with PI (red) and SYBR Green I (green), samples were analyzed for spirochete viability. (A) A single representative image is shown from three biological replicates. Numbers above the images indicate the percentage of dead cells in the standard samples. Scale bars: 10 µm. (B) Percentages of dead spirochetes were measured by fluorescent microscopy and plotted against the known percentage of dead spirochetes per sample. Simple linear regressions were performed using Graphpad Prism 10.6.1. Data are presented as the mean values ± standard deviation from three biological replicates.

**Fig. S4. Mixtures containing live and H_2_O_2_-killed *B. burgdorferi* were analyzed by flow cytometry.** *B. burgdorferi* grown in BSK-II was collected when growth reached the mid-logarithmic phase. Cells were treated with 100 mM H_2_O_2_ for 4h to obtain dead cells. Samples were prepared by mixing proportional live and dead cells. After staining with PI, samples were analyzed for spirochete viability. (A) A single representative image is shown from three biological replicates. Numbers above the images indicate the percentage of dead cells in the standard samples. (B) Percentages of dead spirochetes were measured by flow cytometry and plotted against the known percentage of dead spirochetes per sample. (C) Correlation between measured percent dead values using fluorescent microscopy and flow cytometry. Simple linear regressions were performed using Graphpad Prism 10.6.1. Data are presented as the mean values ± standard deviation from three biological replicates.

**Fig. S5. Sequence alignment of BBI29 to other BbCRASP-1 domain containing proteins.** BBI29 was aligned to CspA, BBE31, BBA66, and BBA69 using the Clustal Omega Multiple Sequence Alignment Tool. CspA residues important for binding host complement regulators FHL-1 and factor H are colored in red.

**Fig. S6. Structural comparison of BBI29 and BbCRASP-1 domain containing proteins.** BBI29 structure was predicted using AlphaFold and compared using PyMol v4.6.0 with the crystal structures of (A) CspA (PDB: 1W33, Chain A), (B) BBE31 (PDB:6FZE, Chain A), (C) BBA66 (PDB: 5IZT, Chain A), and (D) BBA69 (PDB: 6QO1, Chain A). BBI29 is colored green and all other proteins are colored in blue. RMSD scores are labelled in white.

**Fig. S7. Plasmid content of *B. burgdorferi* was determined using multiplex PCR.** (A) lp, linear plasmid; (B) cp, circular plasmid. Lane 1, WT OY617; lane 2, negative control; lane 3, the *bbi29* mutant.

**Table S1. Oligonucleotide primers used in this study.**

| **Primer** | **Target** | **Sequence (5’- 3’)** |
| --- | --- | --- |
| ZM666F | pUC19 Ori Cloning | GTGTAAAGCCTGGGGTGCC |
| ZM666R | pUC19 Ori Cloning | AGGTGCCTCACTGATTAAGCATTG |
| ZM667F | *pflaB-Kan* Cloning | AGATCTCAGCTTTTTTTTGA |
| ZM667R | *pflaB-Kan* Cloning | TCATGAGGCGCGCCG |
| CW03.1F | ∆*bbi29* F2 Arm | ATCAGTGAGGCACCTTAGCTGTTTCTATTG |
| CW03.1R | ∆*bbi29* F2 Arm | AAAAAGCTGAGATCTTCAGGAACCTATTT |
| CW04.1F | ∆*bbi29* F1 Arm | CGGCGCGCCTCATGAGCTTCAGCTTCA |
| CW04.1R | ∆*bbi29* F1 Arm | CCCCAGGCTTTACACATACTAGCACCATT |
| CW06F | *bbi29* | AATTCCTTATAGAGAACAGC |
| CW06R | *bbi29* | CCCGTAGATGATACAAAC |
| ZM68F | *Kan* | TATGCCTCTTCCGACCATCAAGCA |
| ZM68R | *Kan* | AGGCAGTTCCATAGGATGGCAAGA |
